# Decoding heart failure subtypes with neural networks via differential explanation analysis

**DOI:** 10.1093/bib/bbaf581

**Published:** 2025-11-12

**Authors:** Mariano Ruz Jurado, David Rodriguez Morales, Elijah Genetzakis, Fatemeh Behjati Ardakani, Lukas Zanders, Ariane Fischer, Florian Buettner, Marcel H Schulz, Stefanie Dimmeler, David John

**Affiliations:** Institute of Cardiovascular Regeneration, Theodor-Stern-Kai 7, Goethe University Frankfurt, Frankfurt am Main 60590, Hessen, Germany; German Centre for Cardiovascular Research (DZHK), Theodor-Stern-Kai 7, Frankfurt am Main 60590, Germany; Cardio-Pulmonary Institute (CPI), funded by the German Research Foundation (DFG), Hessen, Germany; Institute of Cardiovascular Regeneration, Theodor-Stern-Kai 7, Goethe University Frankfurt, Frankfurt am Main 60590, Hessen, Germany; German Centre for Cardiovascular Research (DZHK), Theodor-Stern-Kai 7, Frankfurt am Main 60590, Germany; Cardio-Pulmonary Institute (CPI), funded by the German Research Foundation (DFG), Hessen, Germany; Institute of Cardiovascular Regeneration, Theodor-Stern-Kai 7, Goethe University Frankfurt, Frankfurt am Main 60590, Hessen, Germany; Faculty of Medicine and Health, University of Sydney, Camperdown, NSW 2050, Australia; Institute for Computational Genomic Medicine, Goethe University Frankfurt, Theodor-Stern-Kai 7, Frankfurt am Main 60590, Hessen, Germany; Institute of Cardiovascular Regeneration, Theodor-Stern-Kai 7, Goethe University Frankfurt, Frankfurt am Main 60590, Hessen, Germany; German Centre for Cardiovascular Research (DZHK), Theodor-Stern-Kai 7, Frankfurt am Main 60590, Germany; Clinic for Cardiology, University Hospital Frankfurt, Theodor-Stern-Kai 7, Frankfurt am Main 60590, Hessen, Germany; Institute of Cardiovascular Regeneration, Theodor-Stern-Kai 7, Goethe University Frankfurt, Frankfurt am Main 60590, Hessen, Germany; German Cancer Research Center (DKFZ), Heidelberg 69120, Baden-Württemberg, Germany; German Cancer Consortium (DKTK), Goethe University Hospital, Frankfurt am Main 60590, Hessen, Germany; German Centre for Cardiovascular Research (DZHK), Theodor-Stern-Kai 7, Frankfurt am Main 60590, Germany; Cardio-Pulmonary Institute (CPI), funded by the German Research Foundation (DFG), Hessen, Germany; Institute for Computational Genomic Medicine, Goethe University Frankfurt, Theodor-Stern-Kai 7, Frankfurt am Main 60590, Hessen, Germany; Institute of Cardiovascular Regeneration, Theodor-Stern-Kai 7, Goethe University Frankfurt, Frankfurt am Main 60590, Hessen, Germany; German Centre for Cardiovascular Research (DZHK), Theodor-Stern-Kai 7, Frankfurt am Main 60590, Germany; Cardio-Pulmonary Institute (CPI), funded by the German Research Foundation (DFG), Hessen, Germany; Institute of Cardiovascular Regeneration, Theodor-Stern-Kai 7, Goethe University Frankfurt, Frankfurt am Main 60590, Hessen, Germany; Cardio-Pulmonary Institute (CPI), funded by the German Research Foundation (DFG), Hessen, Germany

**Keywords:** heart failure subtypes, deep neural networks, explainable artificial intelligence, differential gene expression

## Abstract

Single-cell transcriptomics offers critical insights into the molecular mechanisms of heart failure (HF) with reduced or preserved ejection fraction. However, understanding these mechanisms is hindered by the growing complexity of single-cell data and the difficulty in unmasking meaningful differential gene signatures among HF types. Machine learning, particularly deep neural networks (NNs), address these challenges by learning transcriptional patterns, reconstructing expression profiles and effectively classifying cells but often lacks interpretability. Recent advances in explainable AI (XAI) offer tools to clarify model decisions. Yet pinpointing differentially regulated genes with these tools remains challenging. We introduce a novel method to identify differentially explained genes (DXGs) based on importance scores derived from custom-built NNs. We highlight the superiority of DXGs in identifying HF subtypes-specific pathways that provide new insights into different types of HF. Offering a robust foundation for future research and therapeutic exploration in expanding transcriptome atlases.

## Introduction

Despite the implementation of secondary preventative therapies, cardiovascular diseases remain the leading cause of morbidity and mortality in the aging society [1]. Reliable biomarkers are crucial for early detection of heart failure (HF) and facilitate timely diagnostics and personalized treatment strategies. However, the complexity of the underlying transcriptomic alterations, compounded by the different pathophysiological mechanisms and inter-individual heterogeneity of each disease, is a major challenge for detecting biomarkers and for understanding and distinguishing different types of HF [2, 3]. Large-scale single-nuclei sequencing (snRNA-seq) is widely used to discover biomarkers and understand disease mechanisms that contribute to hypertrophic HF induced by severe aortic valve stenosis (AS), HF with reduced or preserved ejection fraction (HFrEF and HFpEF, respectively) [4–9].

Recent advancements in computational power, and machine learning approaches have offered an unbiased framework to analyse scRNA-seq data [10]. Despite the noise in large transcriptome data caused by data sparsity, biological variability, technical artifacts, and sequencing errors [11, 12], computational methods have shown accurate performance on a variety of tasks, such as, cell type annotation [13], imputation [14] and batch aware data integration [15]. Two particularly successful machine learning approaches are denoising autoencoder (DAE) [16] and deep feed-forward neural networks (NNs). The latter demonstrated capability of precisely classifying transcriptomic data into various biological categories and suggest disease treatments [17, 18].

Taking advantage of DAEs and feed-forward NNs we devised an approach to identify HF related targets and biomarkers by detecting disease-relevant patterns. Our NN was designed to assign each cell a multiclass label that describes its species, cell type, and disease state, only from snRNA-SEQ data.

In addition, we suggest a new statistical approach to investigate which gene expression patterns were important for the model’s decision using Shapley values [19], an approach from explainable AI (XAI). Shapley contribution scores have recently been used in combination with scRNA-seq data to study regulation at the gene-level [20] and for validation purposes for computational methods regarding cell type annotation in murine cardiac hearts [21]. Here we advance this methodology, by designing a statistical test tailored for Shapley gene contribution scores from NNs, which provides an alternative approach for biomarker identification using a concept we term differentially explained genes (DXGs). We provide evidence that DXG derived biomarkers are more reliable than results from classical differential expressed genes (DEGs) methods.

## Materials and methods

### Data preprocessing

Detailed ethical information is published in the previously submitted manuscripts [4, 5, 22]. All other human tissue protocols were approved by the University Hospital of Mainz. Patients provided informed consent in compliance with the Declarations of Helsinki.

SnRNA-seq results were preprocessed using CellRanger (v7.0.0) with reference genome hg38 and mm10, respectively. Secondary data analysis was conducted by Seurat (v4.3.0) [23]. Barcodes with <300, >6000 genes, or >5% MT content were filtered out. Normalization, scaling, and integration were conducted following the guidelines provided from Seurat. Mouse genes were replaced with one-to-one orthologs via OrthoIntegrate [22]. 16,545 uniquely mapped orthologs were included for model training (Supplementary Table 1). CellTypist (v1.6.3) [13] was used, with the Healthy_Adult_Heart (v1) model for annotation. We carefully re-annotated misclassified clusters manually based on known markers.

### Creating training, validation, and test data

Training and validation sets were created using the normalized expression matrices. For validation, we used a stratified hold-out strategy with random sampling (80% training, 20% validation). We ensured equal representation of all cell types via the R package caret (v6.0–94).

### Dimensionality reduction and denoising autoencoder

As input features for the DAE we used normalised expression values of individual cells. The number of input features can be adjusted to the number of expressed genes in the dataset. In this study we used 16 545 orthologous genes as input features, hence our input layer contained 16 545 neurons.

The cell classification algorithm comprises two machine learning models developed using TensorFlow (v2.13.0) [24]. The first model is an autoencoder for dimensionality reduction and denoising purposes. We performed hyperparameter optimization using Keras Turner’s Hyperband [25] bandit-based algorithm and explored neuron ranges: 4000–6000 (first layer), 2000–3000 (second), and 300–500 (latent space), with 50-neuron steps resulting in three encoding (5000, 2400, 350 neurons) and two decoding layers (5150 and 2200 neurons) as the optimal structure.

In addition, we hypertuned using 100 epochs, batch size 10 240, Adam optimizer, and ReLU activation. A common choice for an autoencoder’s loss function is the mean squared error loss, also referred to as the L2-loss:


$$ L2=\frac{1}{n}\mathop{\sum}\limits_{i=1}^n{\left({y}_i-\hat{y_i}\right)}^2\kern0em $$

Here, $n$ is the number of cells, ${y}_i$ the predicted gene expression for cell $i$ and $\hat{y_i}$ to the actual gene expression for cell $i$. During training, we minimized L2-loss and monitored performance. If no improvement occurred after 25 epochs, the Optimizer’s learning rate (initially 0.001) was reduced by 0.1 until reaching 1 × 10^−7^. Training stopped at 500 epochs or after 50 epochs without improvement.

### Multilayer perceptron for multiclass classification

The normalised expression matrices were denoised and reconstructed using the previously described DAE and passed to a MLP multiclass classifier Hyperparameter tuning resulted in three layers containing 795, 230, and 105 neurons.

The MLP’s 13-neuron classification layer uses sigmoid activation for class probabilities; hidden layers use ReLU, with one-hot encoded labeling of cells. The probability values for the 13 classes were assigned a value of 1 or 0, depending on whether they were above a threshold value of 0.5. We defined our macro F1-loss function as follows:


$$ F{1}_{loss}=1-\frac{\left(2\ast pr\ast rc\right)}{\left( pr+ rc\right)} $$

Here $pr$ stands for the precision calculated by:


$$ pr=\frac{True\ positives}{True\ positives+ False\ positives} $$

And $rc$ represents the recall which was calculated by:


$$ rc=\frac{True\ positives}{True\ positives+ False\ negatives} $$

where $True\ positives$ represents the number of correctly classified samples in that class, $False\ positives$ the number of wrongly associated samples in that class and $False\ negatives$ the number of samples that were not classified into a particular class despite belonging to it.

By minimizing this macro F1-loss function we were guaranteed to reach a maximum F1-score. The F1-score is a well-established metric for scenarios where imbalanced classes are given and since cell types *in vivo* data follow such an imbalance, common accuracy calculations resulted in poor performance for underrepresented cell types. Considering the harmonic mean of precision and recall provides a balanced measure that penalizes extreme values and is therefore more suitable for our *in vivo* data. The Adam optimizer strategy was identical to DAE.

### Evaluation of model performance and benchmarking with other tools and datasets

After training we evaluated the performance of the classifier by predicting species, cell type, and disease for each cell of our retained samples and compared them with the actual labels. We calculated a correct classification rate for each of the 13 classes independently. Additionally, we performed a confusion matrix calculation for the species, cell type, and disease categories and visualized the results using the R package cvms (v1.6.2). Benchmarking was carried out by comparing our NN against LR, XGBoost (each with and without DAE), and the attention-based transformer model scGPT (latent space representation and a fine-tuned version). Three individual LR models were created using the glmnet [26] R package for capturing the species, cell type, and disease for each cell. XGBoost was used with its implementation for multilabel classification using an one-hot encoded representation of labels and expression per cell. ScGPT internal embedding for our datasets were extracted and used with the same MLP structure previously described. Additionally, scGPT was fine-tuned in separate runs to learn the species, cell type, and disease using training and validation data.

To further validate our approach, we analyzed a publicly available single-cell RNA sequencing dataset from Koenig *et al.* [37]. This dataset comprises 27 healthy donors and 18 individuals diagnosed with dilated cardiomyopathy (DCM). Following the methodology described above, we trained a NN using this dataset to classify cell types and distinguish between healthy and DCM samples. We calculated DXGs and DEGs between healthy and DCM samples, focusing on CMs and ECs and performed Gene Ontology (GO) term enrichment analysis on uniquely upregulated genes derived from both DXGs and DEGs in CMs.

### Gene contribution score calculations using Shapley values

For calculating Shapley values for all 13 classes, we used the SHAP Python package [27] (v0.44.0). From the training dataset, 1000 cells were randomly selected as the background distribution. DeepExplainer was initiated with the MLP classifier and the previously defined randomly selected background and used to calculate gene contribution scores for each cell in the test dataset. We excluded mitochondrial genes from being possible predictors. Shapley values were Z-transformed rowwise as follows:


$$ {Z}_i=\frac{x_i-{\mu}_i}{\sigma_i} $$

Here ${x}_i$ corresponds for the Shapley value for a given gene of cell $i$, μ*_i_* is the average of all Shapley values for all genes in cell $i$, and ${\sigma}_i$ is the standard deviation of the Shap values for cell $i$. We took advantage of the beneficial data structure of anndata [28] objects and converted each of the 13 sets of z-transformed Shapley values into an individual object. We then proceeded with concatenating them into one data structure keeping track of their individual class assignment.

### Identifying differentially regulated genes based on Shapley values

We added the ground truth information for each cell based on species, cell type, and disease state to define groups of interest that we want to compare. Considering the rather novel nature of Shapley values, we conducted a Shapiro–Wilk test (Scypi-Package) on gene values per cell to test if they follow a normal distribution. Given the ground truth data stored in the metadata of the AnnData object and the actual ground truth data file, we defined groups of cells corresponding to a given species, cell type, and disease state and compared their Shapley values with a second group of cells of interest. Testing can be done either with the Wilcoxon rank-sum test or the Student’s t-test depending on whether the data is normally distributed or not. Throughout this paper Shapley values followed normal distribution and therefore we always applied the Student’s t-test. *P*-values were FDR adjusted using the Benjamini-Hochberg correction. The fold changes between classes were transformed by taking the average cube root of the value per gene following this equation:


\begin{align*} {FC}^3=&\sqrt[3]{mean\left( Shapley\ values\ in\ class\ A\right)}\\ &-\sqrt[3]{mean\left( Shapley\ values\ in\ class\ B\right)}\end{align*}

This calculation is conducted for each gene found in both class $A$ and class $B$, similar as in traditional DGE analysis. The cube root transformation was defined for negative, zero, and positive values, which can be present in the Z-standardization of data. In addition, it reduces the right skew similar to a logarithmic transformation to base 2.

### Differential gene expression analysis and gene set enrichment analysis

We performed DGE analysis using the Wilcoxon and MAST tests implemented in Seurat with default filtering parameters. For GSEA we performed DGE analysis with nonfiltered gene lists. The score per gene for the analysis was calculated by:


$$ {GSEA}_{score}=\mathit{\operatorname{sign}}(FC)\ast \mathit{\log}10\big( abs\left( FC/{p}_{bin}\right)+1 $$

with $FC$ representing the fold change of the given gene and ${p}_{bin}$ representing a binned *P*-value, which is set to 0.1 if it was lower than 0.05 or otherwise set to 1. This encourages a weighted balance between *P*-values and fold changes, without disregarding the change in expression of the given gene. The GSEA was performed by using the fast gene set enrichment (fgsea) method implemented in clusterProfiler R package (v.4.13.2).

### Benchmarking differential gene expression analysis results

We defined a set of GO terms by identifying cell type-specific keywords from pathway analyses in previously published studies [4, 5]. These keywords were then manually reviewed by medical and scientific staff at our facility to ensure their suitability. The final set of terms, used for parsing relevant GO-Terms is accessible in Supplementary Table 8. Resulting GO-Terms were then compared to the results of the GSEA analysis based on the DGE analysis as well as the list of DXGs using Wilcoxon and t-tests, respectively. Finally, we calculated an F1-score using the found terms from our predefined set as true positives and the remaining terms which are not in the defined sets will be treated as true negatives. Terms which were not found were used as false negatives.

## Results

### Neural network design

We designed a NN to process normalized snRNA-seq datasets from diseased and healthy ventricular samples from both patients and mouse models.

After filtering the biological samples, we obtained 144 677 and 40 205 nuclei from healthy patients and healthy mouse hearts, respectively. 41 689 human and 3594 mice nuclei suffer from AS, 19 330 human and 7046 mice with HFrEF and 1712 human and 13 827 murine nuclei with HFpEF [36].

**Figure 1:**
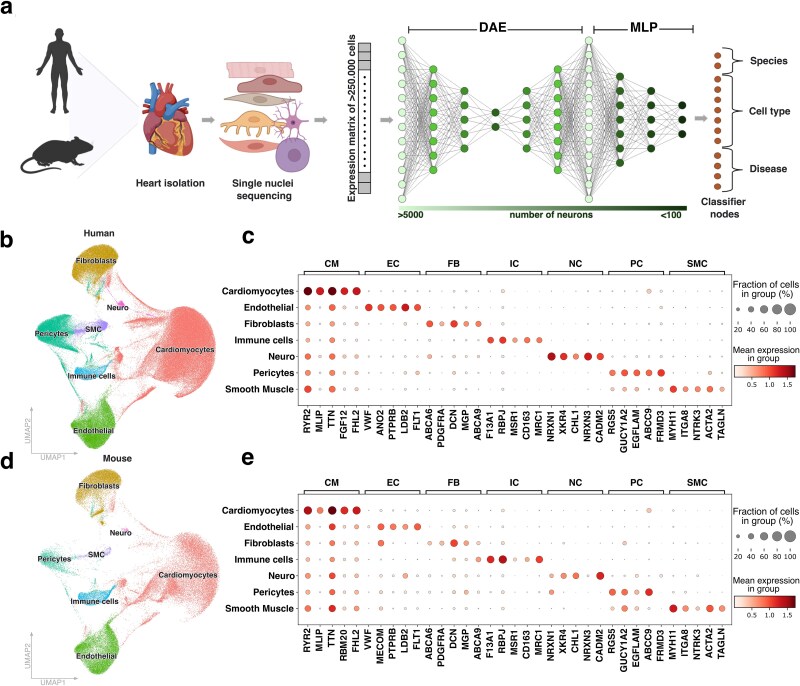
Cell type annotation based on marker genes for deep learning models. (a) schematic overview of workflow. Single nuclei RNA-seq data is obtained from hearts of either diseased or healthy humans and mice. The expression matrix undergoes initial preprocessing before being passed to a denoising autoencoder (DAE) for learning a streamlined representation focused on a narrow bottleneck of the transcriptomic data. Afterwards this representation is used to train a multilayer perceptron, enabling the classification of cells based on species, cell type, and disease state. (b, d) uniform manifold approximation and projection of cell types identified in (b) human and (d) mice. (c, e) average expression and fraction of cells expressing known marker genes for cardiomyocytes (CM), endothelial cells (EC), fibroblasts (FB), immune cells (IC), neuronal cells (NC), pericytes (PC) and smooth muscle cells (SMC) in (c) human and (e) mice.

The integrated dataset included annotations for species, cell type, and disease state, which were encoded in the NN to be learnt as individual classification problems (Fig. 1a). We ensured that our integration and automated cell type annotation was correctly capturing cell clusters by investigating the UMAP per species (Fig. 1b and c, Extended Data Fig. 2c). By inspecting the expression of published cell type-specific markers [5, 37] (Fig. 1d and e), we re-annotated the automated annotation (Extended Data Fig. 3) into cardiomyocytes (CMs), endothelial cells (ECs), fibroblasts (FBs), immune cells (ICs), neuronal cells (NCs), pericytes (PCs) and smooth muscle cells (SMCs).

The NN consists of two components. First, the DAE processes the normalized reads and learns based on a symmetrical layer structure. By reducing the mean squared error loss (L2-loss), a biologically and technically denoised representation of our transcriptome data was generated [16]. Second, a multilayer perceptron (MLP) classifies the nuclei into three distinct classes, differentiating the two species, seven cell types and four disease states. To compensate for imbalanced labels, we defined a custom F1-Loss function based on the harmonic mean of precision and recall. One complete biological sample from each species and disease state was not used for training, guaranteeing a robust performance assessment.

## Network optimization

**Figure 2:**
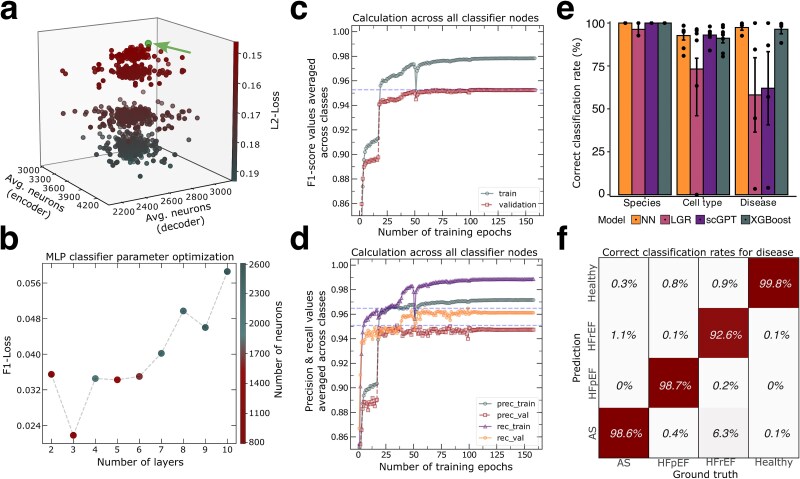
Hypertuned neural network reaches highest accuracies predicting heart failure. (a) three-dimensional scatter plot illustrating the average number of neurons in the decoder and encoder layer of the DEA, along with the corresponding L2-loss for each set of parameters tested during hyperparameter tuning. (b) highlighting the lowest F1-loss values during the hyperparameter tuning of the MLP classifier for each layer configuration. Color indicates the total number of neurons in the network. (c) F1-score calculations per epoch of the MLP classifier’s performance averaged across all classes. (d) precision and recall calculations per epoch of the MLP classifier’s performance averaged across all classes. (e) correct classification rates averaged for species, cell type and disease state. Calculations were made for the custom neural network (NN), logistic regression model (LGR), scGPT and for a XGBoost model. (f) column wise calculations on percentages of correctly classified samples for the classifiers output nodes regarding disease state using the NN.

A Hyperband algorithm was conducted for extensive parameter tuning thus enhancing performance and allowing subsequent biological interpretation of the autoencoder and the MLP components by exploring the layer’s neuron counts (Fig. 2a and b; Extended Data Fig. 4; Methods). As studies indicate that asymmetric neuron designs improved performance of DAE’s [38, 39], we used an asymmetric 3 layered encoder and decoder with 5000, 2400, and 350 neurons and 350, 2200, 5150 neurons, respectively. These neuron counts show the best performance in hypertuning (L2-Loss: 0.1393, Fig. 2a; Extended Data Fig. 4b). For the MLP classifier, a three-layer structure achieved the lowest F1-Loss value (F1-Loss: 0.0217, Fig. 2b; Extended Data Fig. 4c) in parameter hypertuning. Consequently, we used this three-layer network in combination with the best-performing DAE for all subsequent analyses.

### Performance evaluation

By averaging across all classes, we obtained an overall F1-score of 0.9784 (Fig. 2c) for training data and 0.9528 for validation data, indicating a class-unbiased and accurate model structure suitable for downstream model interpretation. By calculating F1-scores separately for species, annotated cell types, and HF conditions, we could exclude potential issues of certain classes being underrepresented during model training. (Extended Data Figs 4d–f).

To determine differences in precision and sensitivity we conducted a separate analysis to examine the precision and recall values (Extended Data Figs 4g–j). Both metrics suggest a strong performance by accurately predicting classes with few false positives, while maintaining high sensitivity and thus minimizing false negatives.

Furthermore, we evaluated the individual misclassifications between categories in the prediction of hold-out samples. To achieve this, we calculated confusion matrices that accurately captured the correct classification rates for each of the 13 attributes. On average our model achieved 100%, 92,7%, and 97,4% accuracy for species, cell type and disease classification, respectively (Fig. 2e, f; Extended Data Fig. 2a, e, i). These high classification rates among all classes allow an in-depth biological interpretation of the MLP classifier decisions.

Training of our NN on an independent dataset from healthy and DCM patients showed that it could successfully classify cell types and diseases with high accuracy (>81.4%), as visualized in Extended Data Fig. 7a and b.

### Comparison to other classification tools

We benchmarked our NN to other single cell classification tools, like a logistic regression (LR) classifier, scGPT [40] and XGBoost [41].

An LR classifier for the three classes (Extended Data Fig. 2) showed that the LR classifier poorly captured the diseases in high dimensional transcriptomic data (Accuracy: 0.6615) showing the need for a complex classification approach. Although the performance slightly improved when we additionally applied our DAE (Extended Data Fig. 2b, f, j). ScGPT and XGBoost were able to classify the species and cell types almost as well as our NN, with slight differences in certain cell types (Extended Data Figs 2c and d, g–h), Yet both tools did not exert comparable performance to our NN in classifying the disease category. XGBoost was able to classify the diseases almost as well as our NN (Accuracy: XGBoost: 0.96, NN: 0,97), but performed worse due to the DAE (Extended Data Fig. 2i). Additional F1-score correlation analysis showed that the NN significantly outperformed other models (Extended Data Figs 2m–o).

### Explainable AI analysis using Shapley values identifies genes that serve as predictors for species, cell types, and heart failure conditions

**Figure 3:**
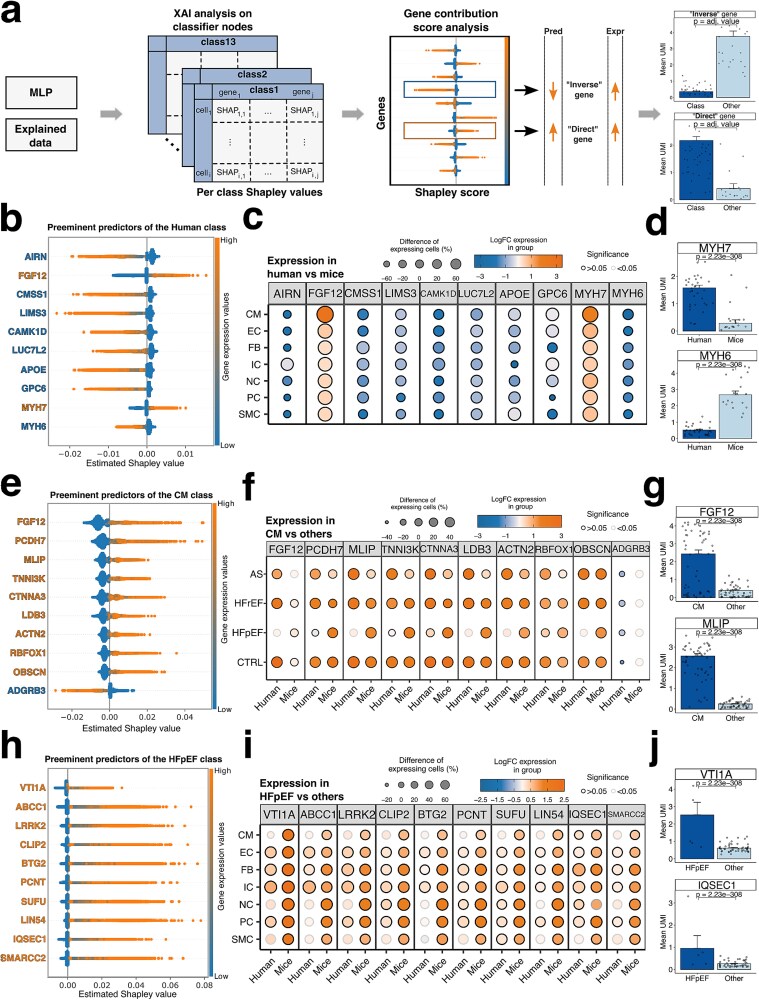
XAI analysis reveals known and potential markers relevant for species, cell types, and heart failure. (a) schematic overview of Shapley values obtained by analyzing the hidden layers of the MLP classifier. Each classifier node produces a set of values corresponding to the gene’s contribution in that class. Prediction and expression are directly correlating or following an inverse relationship based on the type of predictor. Inverse markers show negative Shapley values and are associated with an absence of expression, while direct marker expressions are correlating with positive values. (b, e, h) 10 most contributing predictors visualised by a beeswarm plot for (b) human cells, (e) CMs, and (h) HFpEF cells. An orange gene name indicates a positive correlation between expression and prediction for that class, while a blue gene name indicates a negative correlation. (c, f, i) dot plot illustrating the log fold change in gene expression and the percentage change of cells expressing the indicated genes. Panels represent the following (c) human predictors by cell type across all disease states, (f) CM predictors by disease and species and (i) HFpEF predictors by cell type and species. (d, g, j) averaged unique molecular identifiers (UMI) for two selected genes for (d) human cells against mice cells, (g) CMs against all other remaining cell types, (j) HFpEF cells against the remaining conditions. Statistical significance was determined using the Wilcoxon test on single cell expressions.

In order to identify genes that contribute to the classification performance, we estimated local attribution scores for each cell and each class using Shapley values (Fig. 3a). This approach used the gene expression of 200 cells randomly selected from each cell type in each biological sample. We obtained a set of Shapley values corresponding to the explained data for each of the 13 classification categories covering 52 925 cells and 16 545 genes per category.

The Shapley values for the human and mouse categories exhibit contrasting patterns owing to the binary nature of the decision task, where only one of two outcomes can be realized. Consequently, gene predictors contributing to one of them will obtain opposing scores for the other category. For example, high Shapley values for the myosin heavy chain genes *MYH7/Myh7* were identified as key predictors for differentiating between human and mouse cells (Fig. 3b), showing a direct correlation with its high expression levels in human cells (Fig. 3c and d). Conversely, genes *MYH6/Myh6* exhibited an inverse relationship with the classifier’s predictions, where high expression levels of *MYH6/Myh6* corresponded to lower prediction values for cells associated with humans. Notably, when examining the classification node representing mouse cells, we observed opposite outcomes (Extended Data Fig. 5a, Fig. 3d) consistent with the known difference in myosin heavy chain switch in humans versus mice [42, 43].

Additionally, we found that the long noncoding RNA, *AIRN*, exhibited a pattern similar to that of *MYH6*. High expression levels were associated with cells from mice hearts, while low expression corresponded to human patients (Figs 3b–d). Indeed, *AIRN* has been identified as the largest known imprinted domain in mice, but not in humans [44, 45]. Capturing these well conserved yet differential expressed marker genes among the top predictors for the human/mouse class demonstrates capacity to explore relevant gene markers between both species.

While investigating the top predictors for the CM class, we identified several well-known cell-type markers, including *FGF12*, MLIP, and *TNNI3K* (Fig. 3e). Additionally, we discovered previously unknown predictors that serve as valuable markers for the identification of CMs. For instance, high expression levels of the protocadherin family member 7 (*PCDH7*) and the cell–cell adhesion protein *CTNNA3* were crucial for identifying CMs (Fig. 3e). On the other hand, the model was able to capture down-regulated markers. For instance, the absence of the secretin receptor family member *ADGRB3* was a significant negative predictor (Fig. 3e). The top predictors for the other cell types, along with their expression, are summarized in Extended Data Fig. 5.

### Shapley values reveal novel HFpEF biomarkers

Cardiac-specific biomarkers are crucial for distinguishing between the different HF etiologies, allowing the stratification of patients into risk categories for early diagnosis and targeted intervention. We applied Shapley value analysis on the classifier nodes to predict whether a cell corresponds to AS, HFrEF, or HFpEF (Extended Data Fig. 6, Figs 3h–j). We identified several novel biological candidates across cell types that were crucial for predicting HFpEF. Two exemplary genes are VTI1A and ABCC1 which were top markers for HFpEF and were also significantly upregulated with DXG analysis in CMs but were not significant with traditional DEG analysis. The gene *VTI1A* emerged as a key predictor for cells in HFpEF, representing a novel candidate for patients with this condition. While its role in the heart is understudied, one study linked *VTI1A* variants to altered length of the QRS complex, pointing towards a role in CM depolarization. Notably, prolonged QRS-intervals have been associated with impaired prognosis in HFpEF patients [46, 47].

*ABCC1*, also referred to as *MRP1*, is a membrane-bound transporter that facilitates the extracellular efflux of both endogenous and exogenous molecules from cells, playing a key role in cellular detoxification and drug resistance mechanism [48–50]. Notably, *ABCC1* mediates the removal of cyclic GMP-AMP (cGAMP) [49], and in mice, elevated cGAMP levels were observed to impair vascular remodelling, and EC proliferation [51]. Furthermore, heightened cGAMP serum concentrations were observed in HF patients [52], reinforcing the potential importance of this candidate.

*LRRK2* encodes a multidomain protein involved in intracellular processes like microtubule dynamics and vesicular trafficking [53]. While mutations in *LRRK2* are well-known in Parkinson’s disease [54], its role in HFpEF is largely unexplored. However, a recent study reported a negative correlation between circulating levels of *LRRK2* with NT-proBNP, a biomarker for HF [55]. In mice, *LRRK2* expression was found to be upregulated in cardiac ECs under HFpEF conditions [56]. Furthermore, *LRRK2* knockout mice subjected to TAC exhibited improved cardiac function and reduced adverse remodelling compared to their WT counterparts. These beneficial effects were found to be mediated through autophagy-related processes [57]. Although other studies have also confirmed a role for *LRRK2* in monocyte adhesion to ECs via NF-κΒ [58], a distinct requirement for the activation of sterile inflammation in HFpEF [59]. Each of these novel molecular targets could lead to effective detection of HFpEF, with implications for improving disease prognosis and management.

### Differential explanation analysis

**Figure 4:**
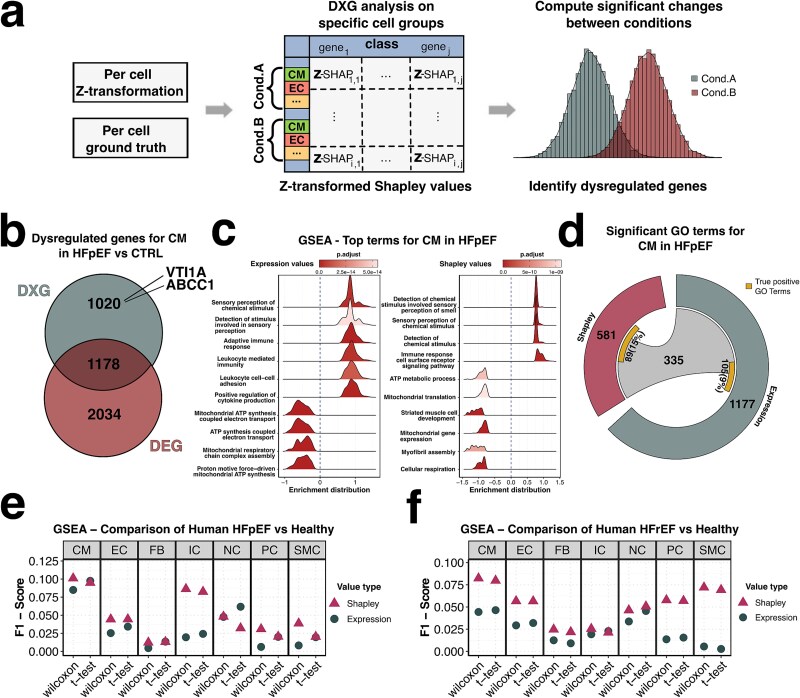
Exchanging expression values for Shapley contribution scores enhances the results of pathway analysis. (a) overview of the process for DXG analysis using Shapley values. First, the contribution scores are Z-transformed for each gene and combined with the ground truth data to identify relevant groups of cell types and conditions for comparison. The Shapley value sets for both conditions are then statistically tested to determine differences and identify dysregulated genes between the conditions. (b) comparison of the overlap of dysregulated genes identified in CM under the HFpEF condition versus the CTRL condition. DXGs were calculated by applying a t-test to Z-transformed Shapley values and DEGs were calculated through a Wilcoxon test applied on expression values. Two genes found exclusively in DXGs for patient CM under the HFpEF condition are named (c) top 10 GSEA terms for CM under the HFpEF condition ranked by enrichment score, based on statistics from Wilcoxon test using expression values (left) and using the t-test applied to Shapley values (right). *P*-values were corrected using the Benjamini-Hochberg correction. (d) significant GO terms for CM under the HFpEF condition identified through GSEA analysis are shown for Shapley values (left circular part) and expression values (right circular part). A chord highlights the terms shared between both analyses. Additionally, a yellow box displays the number of true positive GO terms for HFpEF in CM, illustrating the proportions found uniquely and commonly by each method. (e and f), F1-score calculations for GSEA results, performed separately using the Wilcoxon test and the t-test, are shown for each cell type. These calculations are based on expression values and Shapley values in (e) the HFpEF condition and (f) the HFrEF condition.

We then considered a method to statistically test whether Shapley values between two groups of cells show significant variations. Since these values were derived from transcriptomic data that reflect the gene expression values, we employed them to identify dysregulated genes among groups of interest, similar to traditional differential gene expression (DGE) analysis. Using the original labels per cell, we performed Student’s t-test to identify significant differences in Shapley values per gene in each group (Fig. 4a, Supplementary Tables 3–6). Since we were no longer calculating DEGs based on expression levels alone, but instead using XAI, which reflects the predicted importance derived from the NN, we refer to these genes as DXGs. To evaluate whether our lists of DXGs capture different sets of genes compared to the traditional Wilcoxon test on expression data, we first assessed the overlap and differences between DXGs and DEGs identified in CMs from HFpEF patients (Fig. 4b, Supplementary Table 7). Using a t-test on Shapley values comparing healthy-labeled CMs to HFpEF-labeled CMs we identified 2198 dysregulated genes while the Wilcoxon test on expression values identified 3212 genes. Of these, 1178 genes were common to both methods (Supplementary Table 3 and 7). These findings demonstrate that each approach identifies unique genes that may be relevant biomarkers for HFpEF, although there is a significant overlap between the DXGs and DEGs.

Next, we investigated whether the differences in identified genes led to the detection of distinct mechanisms associated with HFpEF in CMs. To explore this, we performed gene set enrichment analysis (GSEA) using *P*-values calculated for each gene with both methods (Fig. 4c, Methods). While both approaches highlighted similar significant terms, such as those related to sensory stimuli and ATP metabolic processes, DXG analysis also revealed additional terms relevant to CMs under HF conditions. Notably, one of the most regulated terms identified through this method pertained to muscle cell development (Fig. 4c).

We then assessed the differences in identified pathways by comparing the significant pathways obtained from both methods and referencing them against a predefined set of true positive pathways known to be dysregulated in HF-affected CMs (Fig. 4d, Supplementary Table 8). The GSEA based on Shapley values identified 581 significant pathways, while the analysis using expression values yielded 1177 terms, with 335 pathways overlapping between the two methods (Supplementary Table 9). Comparing these pathways to our true positive list, we found that 15% of the terms identified with Shapley values matched the predefined pathways, whereas only 9% of the terms from expression values matched. This indicates that the expression-based results are more prone to noise and the inclusion of irrelevant terms, potentially due to the sparsity of expression data (Figs 4d–f).

On an independent and previously published dataset from Koenig *et al.* [37]. Our DXG analysis identified the majority of genes previously reported as critical targets in the original study (Extended Data Fig. 7d and e). GO-term enrichment analysis revealed that uniquely upregulated genes from the DXG analysis in CMs were significantly associated with HF and cardiac muscle-related processes (highlighted in red in Extended Data Fig. 7f). In contrast, the DEG analysis yielded less specific functional annotations.

To determine whether Shapley values consistently outperform expression values, we conducted GSEAs using both value types in combination with Wilcoxon and t-tests, repeating the analysis across all cell types and.

(Fig. 4e and f, Supplementary Table 9 and 10). We then calculated F1-scores by comparing the identified pathways to our previously defined list of true positives. The results demonstrated that Shapley values consistently outperformed expression values, further emphasizing their superiority in detecting dysregulated genes and their associated pathways.

## Discussion

In this study, we present a novel approach combining a DAE with a MLP and an adjusted loss function to classify species, cell types, and heart diseases based on scRNA-SEQ data. This strategy demonstrates the sufficiency of individual transcriptomes to detect various HF types (HFpEF and HFrEF) in mice and humans with high accuracy (F1-scores of >0.95).

When combined with XAI tools, specifically Shapley values, these models have the potential to significantly enhance unbiased biomarker discovery in cardiovascular research. In this pilot study, our model captured complex transcriptomic patterns, such as species-specific expression of myosin heavy chain genes (*MYH7* and *MYH6*), thus validating the model’s biological interpretability. Importantly, this analysis reaffirmed additional known interspecies differences while uncovering novel insights, such as the role of *AIRN* in distinguishing mouse and human samples, underscoring the potential of our approach for identifying key markers of cardiac phenotypes.

Shapley values have first been introduced by Lundenberg *et al.* [27] and since then have been used to predict cell type markers from single-cell data in early developmental stages [29], identify severe risk factors for cardiovascular diseases [30] or pinpoint disease associated enhancers [20]. Here, our multiclass MLP model uniquely demonstrates that Shapley values can identify biomarkers for cell types, species, and diseases from a single MLP model that was trained with scRNA-SEQ data.

Our exploration of hidden layers enabled the identification of predictive genes for species, cell types, and HF conditions. Novel findings, such as the implication of *VTI1A* and *ABCC1* in HFpEF, provide valuable leads for further research, especially given their links to processes like vesicular trafficking and immune regulation, which may play a crucial role to drive immunological alterations in HF [31, 32]. Furthermore, our model effectively captured established cardiac markers, while revealing potential novel markers, such as *CTNNA3* and *PCDH7* for CMs, thus advancing cell type annotation and phenotypic stratification.

By calculating DXGs, we proposed a novel method for identifying dysregulated genes and biomarkers. In contrast to DEGs, which only consider the gene expression under two conditions, the comparison of Shapley values considers all possible permutations of genes and ranks their importance across all possible subsets of genes. We hypothesize that this approach reduced the number of irrelevant DEGs potentially caused by data sparsity or other confounding factors. Yielding more targeted and reliable disease-specific markers. Shapley values or similar contribution scores in general could provide a robust alternative to traditional statistical methods by integrating the advantages of machine learning techniques to overcome the limitations of traditional DEG analysis, particularly in sparse and noisy datasets.

The robustness of our NN could be demonstrated by calculating DXGs on a previously published dataset from healthy individuals and DCM patients. We were able to retain disease-related marker genes, while genes identified solely through DXG analysis exhibited enrichment terms related to heart disease and CMs.

Despite the promising results, high computational costs, small sample size and restriction to ortholog genes between humans and mice may limit the depth of biological insights [22]. While our classifier showed reasonable reliability and outperformed LR, XGBoost, and scGPT in classifying cell types and diseases, it still could require refinements that could reduce bias and improve Shapley value accuracy. Additionally, transformer based approaches with huge cell atlases show promising results and should be compared to our method [33]. In order to compare DEG and DXG analysis results we used previously published and manually validated “true positive” GO-Terms. Although we have tried to be as neutral as possible in choosing GO-Terms that are relevant to the cell types in the corresponding HFs, manual selection is not error-free and could potentially contain keywords that are not relevant to the disease.

To address these limitations, future efforts should focus on increasing sample sizes, incorporating more diverse datasets, and exploring emerging machine learning frameworks. Especially the future integration and training with large organ cell atlases [5, 34, 35] could further prove the robustness and the clinical importance of the genes detected by our established method.

The introduction of DXGs as a concept adds a powerful dimension to traditional DGE analysis. By demonstrating a higher signal-to-noise ratio and increased precision in identifying pathways relevant to HF phenotypes, our approach highlights the added value of incorporating machine learning-based feature attribution in transcriptomic studies. This methodology may outperform traditional expression-based analyses in uncovering biologically relevant pathways. Future studies are essential to address if the identified genes using this novel approach indeed have biologically validated functions to ultimately document the value of the developed tool.

## Conclusion

Our work underscores the potential of advanced NN architectures integrated with XAI to enhance our understanding of cardiac biology. The ability to reliably classify cell types and disease states while identifying key predictive genes and pathways positions this framework as a transformative tool in cardiac research. Future studies could leverage this approach to investigate other complex biological systems, ensuring broader applicability and deeper insights into disease mechanisms.

**Key Points**

- We propose Differentially Explained Genes (DXGs) – a new concept leveraging on Shapley values from explainable AI (XAI) to identify genes most relevant for characterizing conditions.
- In a case study, we integrated scRNA-seq data from human patients and mouse models, revealing novel candidate genes most relevant for heart failure.
- The successful classification of subtypes of heart failures in different species and the detection of shared and species-specific gene expression patterns demonstrates the robustness and translational potential of DXGs.
- Gene set enrichment analysis using DXGs detected a higher proportion of biologically validated pathways related to heart failure than expression-based GSEAs.
- We conclude that DXGs provide more interpretable and biological meaningful insights than traditional DEG methods, and may serve as an innovative tool for biomarker discovery.

## Supplementary Material

Suppl_Figures_Revision1_bbaf581(1)

## Data Availability

Human healthy heart (left ventricle and septum) data was obtained through the Human Cell Atlas (HCA) (https://explore.data.humancellatlas.org/projects/). Human and mice HFrEF is available at ArrayExpress (E-MTAB-13264 and E-MTAB-7869). Hypertrophic hearts were obtained from Nicin *et al.* [4] (E-MTAB-11268) and mice treated with HFD/L-NAME were obtained by Kattih *et al.* [56] (E-MTAB-14589). Human HFpEF and mice TAC samples were uploaded to arrayexpress (E-MTAB-14753). The additional DXG analysis was performed on a public dataset from Koenig *et al.* [37] comprising DCM patients and healthy controls, available at the Gene Expression Omnibus (GSE183852). Training and test labels with their features were uploaded to Huggingface (https://huggingface.co/datasets/mruzjurado/CVD_dataset/tree/main). All source code is available at https://github.com/MarianoRuzJurado/RuzJurado_et_al_2025.

## References

[1] Roth GA, Mensah GA. Global burden of cardiovascular diseases and risk factors, 1990-2019: update from the GBD 2019 study. J Am Coll Cardiol 2020;76:2982–3021. 10.1016/j.jacc.2020.11.010. 33309175 PMC7755038

[2] Rosch S, Rommel K-P, Scholz M. et al. Transcriptomic research in heart failure with preserved ejection fraction: current state and future perspectives. Card Fail Rev 2020;6:e24. 10.15420/cfr.2019.19. 33042584 PMC7539142

[3] Simmonds SJ, Cuijpers I, Heymans S. et al. Cellular and molecular differences between HFpEF and HFrEF: a step ahead in an improved pathological understanding. Cells 2020;9:242. 10.3390/cells9010242. PMC7016826 31963679

[4] Nicin L, Schroeter SM. A human cell atlas of the pressure-induced hypertrophic heart. Nat Cardiovasc Res 2022;1:174–85. 10.1038/s44161-022-00019-7. 39195989 PMC11357985

[5] Litviňuková M, Talavera-López C. Cells of the adult human heart. Nature 2020;588:466–72. 10.1038/s41586-020-2797-4. 32971526 PMC7681775

[6] Tombor LS, John D. Single cell sequencing reveals endothelial plasticity with transient mesenchymal activation after myocardial infarction. Nat Commun 2021;12:681. 10.1038/s41467-021-20905-1. 33514719 PMC7846794

[7] Du L, Sun X, Wang T. et al. Single cell and lineage tracing studies reveal the impact of CD34+ cells on myocardial fibrosis during heart failure. Stem Cell Res Ther 2023;14:33. 10.1186/s13287-023-03256-0. 36805782 PMC9942332

[8] Lee SH, Kim N. Single-cell transcriptomics reveal cellular diversity of aortic valve and the immunomodulation by PPARγ during hyperlipidemia. Nat Commun 2022;13:5461. 10.1038/s41467-022-33202-2. 36115863 PMC9482653

[9] Li G, Zhao H. Single-cell transcriptomic profiling of heart reveals ANGPTL4 linking fibroblasts and angiogenesis in heart failure with preserved ejection fraction. J Advert Res 68:215–30. 10.1016/j.jare.2024.02.006. PMC11785561 38346487

[10] Liu J, Fan Z, Zhao W. et al. Machine intelligence in single-cell data analysis: advances and new challenges. Front Genet 2021;12:655536. 10.3389/fgene.2021.655536. 34135939 PMC8203333

[11] Kim JK, Kolodziejczyk AA, Ilicic T. et al. Characterizing noise structure in single-cell RNA-seq distinguishes genuine from technical stochastic allelic expression. Nat Commun 2015;6:8687. 10.1038/ncomms9687. 26489834 PMC4627577

[12] Kolodziejczyk AA, Kim JK, Svensson V. et al. The technology and biology of single-cell RNA sequencing. Mol Cell 2015;58:610–20. 10.1016/j.molcel.2015.04.005. 26000846

[13] Domínguez Conde C, Xu C. Cross-tissue immune cell analysis reveals tissue-specific features in humans. Science 2022;376:eabl5197. 10.1126/science.abl5197. 35549406 PMC7612735

[14] Arisdakessian C, Poirion O, Yunits B. et al. DeepImpute: an accurate, fast, and scalable deep neural network method to impute single-cell RNA-seq data. Genome Biol 2019;20:211. 10.1186/s13059-019-1837-6. 31627739 PMC6798445

[15] Lopez R, Regier J, Cole MB. et al. Deep generative modeling for single-cell transcriptomics. Nat Methods 2018;15:1053–8. 10.1038/s41592-018-0229-2. 30504886 PMC6289068

[16] Eraslan G, Simon LM, Mircea M. et al. Single-cell RNA-seq denoising using a deep count autoencoder. Nat Commun 2019;10:390. 10.1038/s41467-018-07931-2. 30674886 PMC6344535

[17] Zhao X, Wu S, Fang N. et al. Evaluation of single-cell classifiers for single-cell RNA sequencing data sets. Brief Bioinform 2020;21:1581–95. 10.1093/bib/bbz096. 31675098 PMC7947964

[18] Alharbi F, Vakanski A. Machine learning methods for cancer classification using gene expression data: a review. Bioengineering (Basel) 2023;10:173. 10.3390/bioengineering10020173 PMC9952758 36829667

[19] Shapley, L. S . 17. A value for n-person games. in *Contributions to the Theory of Games (AM-28), Volume II* (eds. Kuhn, H. W. & Tucker, A. W.) 307–18 Princeton University Press, Princeton, 1953, 10.1515/9781400881970-018.

[20] Mitra S, Malik R. Single-cell multi-ome regression models identify functional and disease-associated enhancers and enable chromatin potential analysis. Nat Genet 2024;56:627–36. 10.1038/s41588-024-01689-8. 38514783 PMC11018525

[21] Galdos FX, Xu S. devCellPy is a machine learning-enabled pipeline for automated annotation of complex multilayered single-cell transcriptomic data. Nat Commun 2022;13:5271. 10.1038/s41467-022-33045-x. 36071107 PMC9452519

[22] Jurado MR, Tombor LS. Improved integration of single-cell transcriptome data demonstrates common and unique signatures of heart failure in mice and humans. Gigascience 2024;13:giae011. 10.1093/gigascience/giae011. PMC10993718 38573186

[23] Hao Y, Hao S. Integrated analysis of multimodal single-cell data. Cell 2021;184:3573–3587.e29. 10.1016/j.cell.2021.04.048. 34062119 PMC8238499

[24] Developers, T. TensorFlow. ( Zenodo, 2024). 10.5281/ZENODO.4724125.

[25] Li L, Jamieson K, DeSalvo G. et al. Hyperband: a novel bandit-based approach to hyperparameter optimization. J Mach Learn Res 2018;18:1–52.

[26] Friedman J, Hastie T, Tibshirani R. Regularization paths for generalized linear models via coordinate descent. J Stat Softw 2010;33:1–22. 10.18637/jss.v033.i01. 20808728 PMC2929880

[27] Guyon I, Luxburg UV, Bengio S. et al. (eds). A unified approach to interpreting model predictions. In: Advances in Neural Information Processing Systems 30 (NeurIPS 2017). Red Hook, NY: Curran Associates, Inc.; 2017.

[28] Virshup et al. anndata: Access and store annotated data matrices. Journal of Open Source Software 2024;9:4371. 10.21105/joss.04371

[29] Proks M, Salehin N, Brickman JM. Deep learning-based models for preimplantation mouse and human embryos based on single-cell RNA sequencing. Nat Methods 22:207–16. 10.1038/s41592-024-02511-3. PMC11725497 39543284

[30] Luo H, Xiang C. SHAP based predictive modeling for 1 year all-cause readmission risk in elderly heart failure patients: feature selection and model interpretation. Sci Rep 2024;14:17728. 10.1038/s41598-024-67844-7. 39085442 PMC11291677

[31] Mann DL . The emerging field of cardioimmunology: past, present and foreseeable future. Circ Res 2024;134:1663–80. 10.1161/CIRCRESAHA.123.323656. 38843286 PMC11160976

[32] Swirski FK, Nahrendorf M. Cardioimmunology: the immune system in cardiac homeostasis and disease. Nat Rev Immunol 2018;18:733–44. 10.1038/s41577-018-0065-8. 30228378

[33] Theodoris CV, Xiao L. Transfer learning enables predictions in network biology. Nature 2023;618:616–24. 10.1038/s41586-023-06139-9. 37258680 PMC10949956

[34] Regev A, Teichmann SA. Science forum: the human cell atlas. eLife 6:e27041. 10.7554/eLife.27041. PMC5762154 29206104

[35] Sikkema L, Ramírez-Suástegui C, Strobl DC. et al. An integrated cell atlas of the lung in health and disease. Nat Med 2023;29:1563–77. 10.1038/s41591-023-02327-2. 37291214 PMC10287567

[36] Schiattarella GG, Altamirano F. Nitrosative stress drives heart failure with preserved ejection fraction. Nature 2019;568:351–6. 10.1038/s41586-019-1100-z. 30971818 PMC6635957

[37] Koenig AL, Shchukina I. Single-cell transcriptomics reveals cell-type-specific diversification in human heart failure. Nat Cardiovasc Res 2022;1:263–80. 10.1038/s44161-022-00028-6. 35959412 PMC9364913

[38] Huang Z, Jin X, Lu C. et al. Contrastive masked autoencoders are stronger vision learners. IEEE Trans Pattern Anal Mach Intell 2024;46:2506–17. 10.1109/TPAMI.2023.3336525 38015699

[39] Gilbert MS, de Campos MLR, Campista MEM. Asymmetric autoencoders: an NN alternative for resource-constrained devices in IoT networks. Ad Hoc Netw 2024;156:103412. 10.1016/j.adhoc.2024.103412.

[40] Cui H, Wang C. scGPT: toward building a foundation model for single-cell multi-omics using generative AI. Nat Methods 2024;21:1470–80. 10.1038/s41592-024-02201-0. 38409223

[41] XGBoost. 10.1145/2939672.2939785.

[42] Hsieh J, Becklin KL. Myosin heavy chain converter domain mutations drive early-stage changes in extracellular matrix dynamics in hypertrophic cardiomyopathy. Front Cell Dev Biol 2022;10:894635. 10.3389/fcell.2022.894635. 35784482 PMC9245526

[43] VanBuren P, Harris DE, Alpert NR. et al. Cardiac V1 and V3 myosins differ in their hydrolytic and mechanical activities in vitro. Circ Res 1995;77:439–44. 10.1161/01.RES.77.2.439. 7614728

[44] Di Michele F, Chillón I, Feil R. Imprinted long non-coding RNAs in mammalian development and disease. Int J Mol Sci 2023;24:13647. 10.3390/ijms241713647. PMC10487962 37686455

[45] Statello L, Guo C-J, Chen L-L. et al. Author correction: gene regulation by long non-coding RNAs and its biological functions. Nat Rev Mol Cell Biol 2021;22:159. 10.1038/s41580-021-00330-4. PMC8095262 33420484

[46] Spielmann N, Miller G. Extensive identification of genes involved in congenital and structural heart disorders and cardiomyopathy. Nature Cardiovascular Research 2022;1:157–73. 10.1038/s44161-022-00018-8. PMC11358025 39195995

[47] Joseph J, Claggett B, Anand I. et al. QRS Duration Is a Predictor of Adverse Outcomes in Heart Failure With Preserved Ejection Fraction. J Am Coll Cardiol HF 2016;4:477–86. 10.1016/j.jchf.2016.02.013. 27039126

[48] Devine K, Villalobos E. The ATP-binding cassette proteins ABCB1 and ABCC1 as modulators of glucocorticoid action. Nat Rev Endocrinol 2022;19:112–24. 10.1038/s41574-022-00745-9. 36221036

[49] Maltbaek JH, Cambier S, Snyder JM. et al. ABCC1 transporter exports the immunostimulatory cyclic dinucleotide cGAMP. Immunity 2022;55:1799–1812.e4. 10.1016/j.immuni.2022.08.006. 36070769 PMC9561016

[50] Jungsuwadee P, Nithipongvanitch R. Mrp1 localization and function in cardiac mitochondria after doxorubicin. Mol Pharmacol 2009;75:1117–26. 10.1124/mol.108.052209. 19233900 PMC2672805

[51] Huang LS, Hong Z. mtDNA activates cGAS Signaling and suppresses the YAP-mediated endothelial cell proliferation program to promote inflammatory injury. Immunity 2020;52:475–486.e5. 10.1016/j.immuni.2020.02.002. 32164878 PMC7266657

[52] Hailati J, Liu ZQ. Increased cyclic guanosine monophosphate and interleukin-1Beta is activated by mitochondrial dysfunction and associated with heart failure in atrial fibrillation patients. Cardiol Res 2024;15:108–16. 10.14740/cr1613. 38645829 PMC11027785

[53] LRRK2 deficiency protects the heart against myocardial infarction injury in mice via the P53/HMGB1 pathway. Free Radic Biol Med 2022;191:119–27. 10.1016/j.freeradbiomed.2022.08.035. 36055602

[54] Neurocirculatory and nigrostriatal abnormalities in Parkinson disease from LRRK2 mutation. Placeholder Text Neurology 2007;69:1580–1584. 10.1212/01.wnl.0000268696.57912.64. 17625107

[55] Azzo JD, Dib MJ. Proteomic associations of NT-proBNP (N-terminal pro-B-type natriuretic peptide) in heart failure with preserved ejection fraction. Circ Heart Fail 17:2. 10.1161/CIRCHEARTFAILURE.123.011146. PMC7615693 38299345

[56] Inhibition of miR-92a normalizes vascular gene expression and prevents diastolic dysfunction in heart failure with preserved ejection fraction. J Mol Cell Cardiol 2025;198:89–98. 10.1016/j.yjmcc.2024.11.004. 39592091

[57] Leucine-rich repeat kinase-2 deficiency protected against cardiac remodelling in mice via regulating autophagy formation and degradation. J Adv Res 2022;37:107–17. 10.1016/j.jare.2021.07.004. 35499056 PMC9039674

[58] Hongge L, Kexin G, Xiaojie M. et al. The role of LRRK2 in the regulation of monocyte adhesion to endothelial cells. J Mol Neurosci 2015;55:233–9. 10.1007/s12031-014-0312-9. 24788225

[59] Deloer S, Fuss I. Inhibition of LRRK2 kinase activity with a novel inhibitor (CS 82) inhibits NLRC4 inflammasome activation and reduces intestinal inflammation severity. J Immunol 2023;210:61.10. 10.4049/jimmunol.210.Supp.61.10.36445376

