# Supplementary material for: Decoding heart failure subtypes with neural networks via differential explanation analysis
Source: Brief Bioinform. 2025 Nov 12;26(6):bbaf581. doi: 10.1093/bib/bbaf581 (PMC12610404; doi:10.1093/bib/bbaf581)
Supplement: Suppl_Figures_Revision1_bbaf581(1) [file suppl_figures_revision1_bbaf581(1).docx]

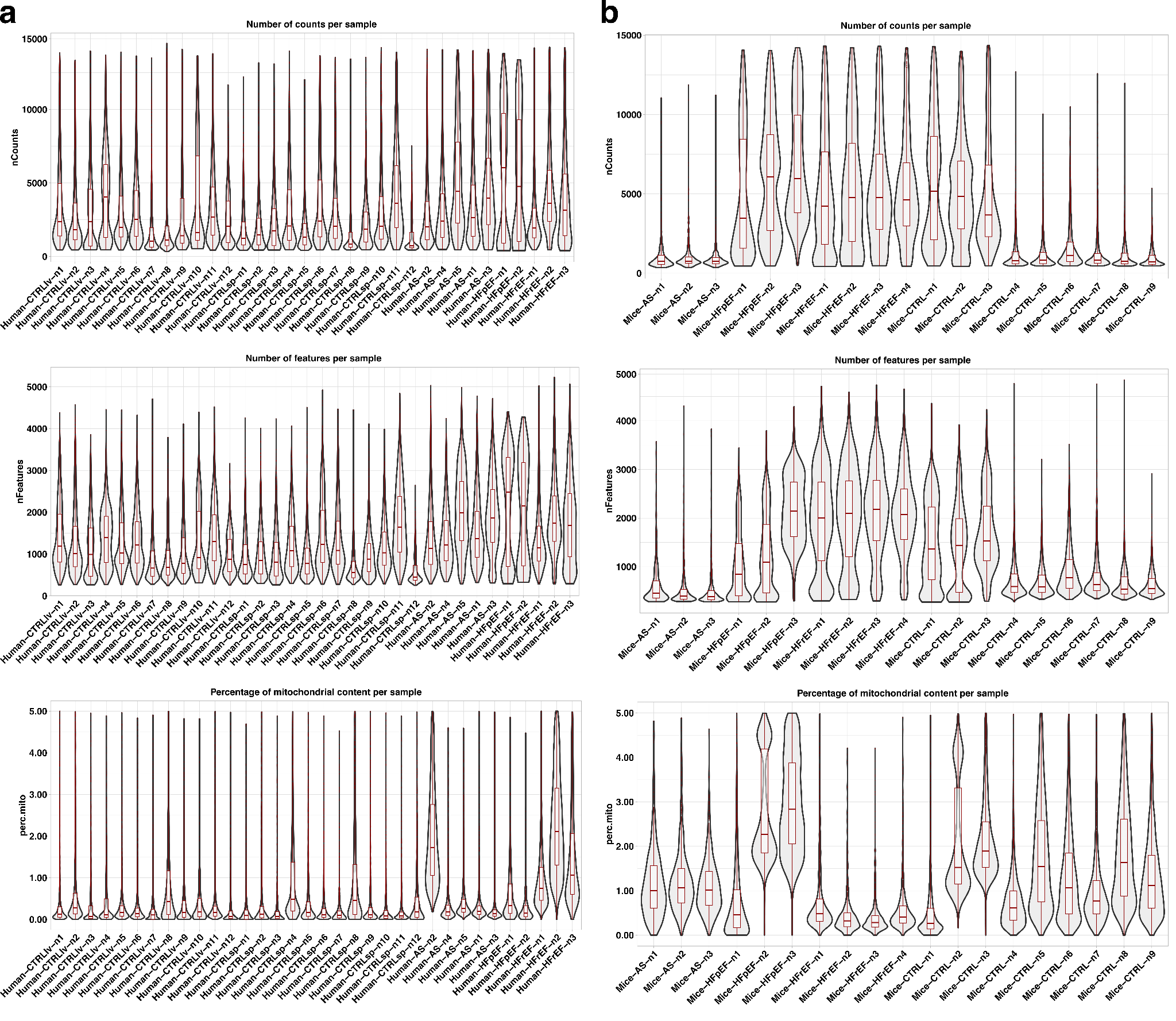


**Extended Data Fig.1: Quality measurements of snRNA-seq cells used for training, validation and test sets.** **a-b**, Number of counts per sample, number of features per sample and percentage of mitochondrial content per sample for each heart failure state obtained in **a)** patients and **b)** mice models.


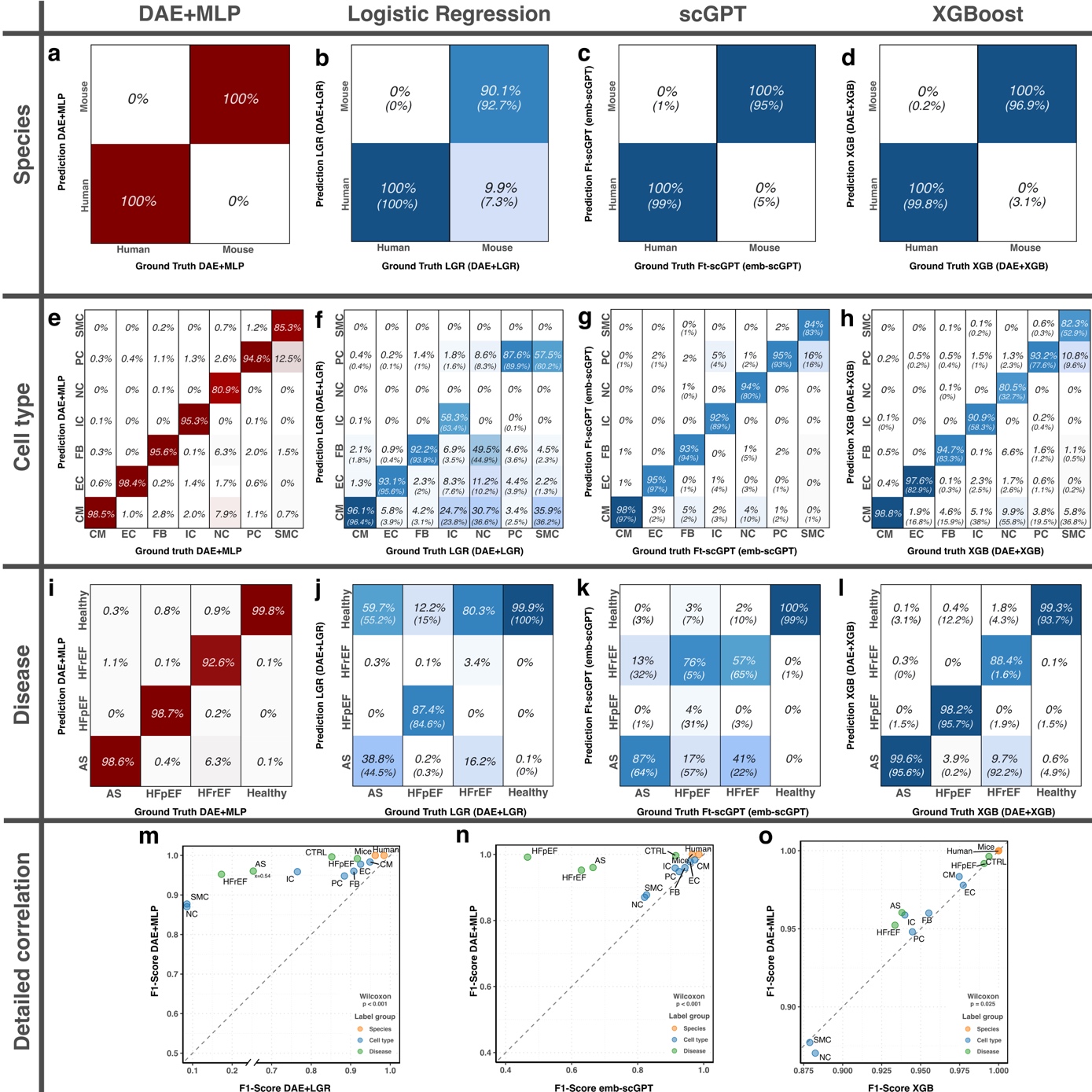


**Extended Data Fig. 2: Benchmarking of the neural network against baseline models.** Correct classification rates are shown for species **a–d**, cell type **e–h**, and disease state **i–l**. Values in parentheses indicate performance when using our DAE or, for scGPT, its latent space representation combined with our MLP. Models compared include: DAE+MLP **(a, e, i)**, logistic regression **(b, f, j)** with (DAE+LGR) and without autoencoder (LGR), scGPT **(c, g, k)** with fine-tuning (Ft-scGPT) or latent embeddings (emb-scGPT) and XGBoost **(d, h, l)** with (DAE+XGB) or without autoencoder (XGB). **m-o,** F1-score correlation analysis for each label for our DAE+MLP against **m)** DAE+LGR, **n)** emb-scGPT and **o)** XGB. Significance test was performed by using a paired Wilcoxon test.


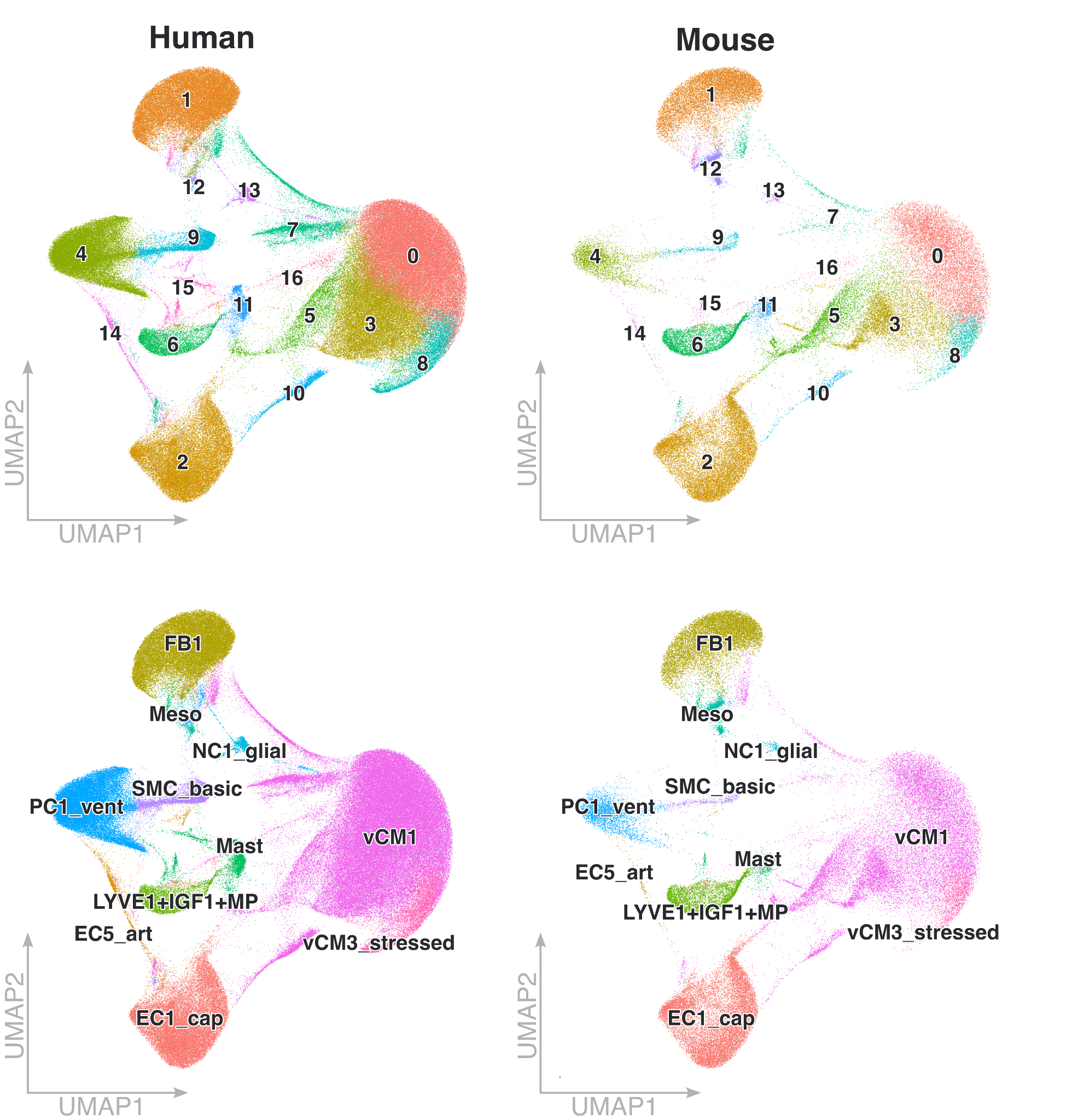


**Extended Data Fig.3: Automatic annotation of unsupervised clustering representing the data.** Automatic annotation of the 16 identified clusters using CellTypist. The identified clusters belong to cardiomyocytes from the ventricle (vCM1, vCM3), capillary and arterial endothelial cells (EC1, EC5, respectively), fibroblasts (FB1), ventricular pericytes (PC1), smooth muscle cells (SMC), cells which show LYVE1 and IGF expression or are macrophages (LYVE1+IFG1+MP), neuronal cells (NC1), mesodermal cells (Meso) and mast cells (Mast).


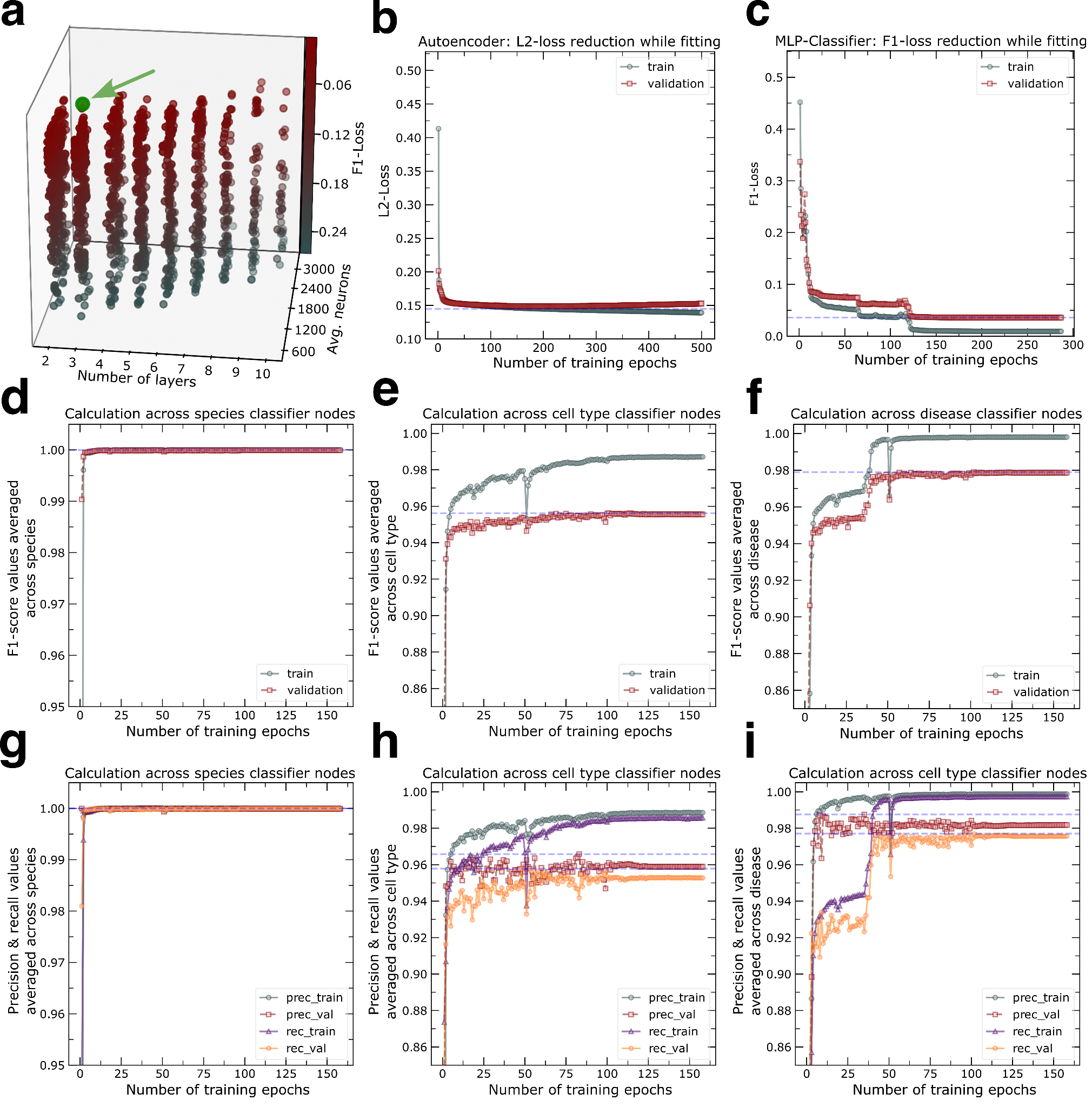


**Extended Data Fig.4: Learning curves of neural networks show little evidence of overfitting but high F1-scores. a**, Three-dimensional scatter plot illustrating the F1-Loss in the MLP classifier layer, along with the corresponding number of neurons for each layer configurations tested during hyperparameter tuning. **b**, L2-loss calculations per epoch of the DAE considering the training data (blue) and the validation data (red). **c**, F1-loss calculations per epoch of the MLP classifier for the training data (blue) and for the validation data (red). **df,** Per epoch F1-score calculation during the classifiers fitting to the heart failure data averaged across **d)** species, **e)** cell type and **f)** disease state. **g-i,** Per epoch precision and recall calculations for the classifier while fitting averaged across **g)** species, **h)** cell type and **i)** disease state.


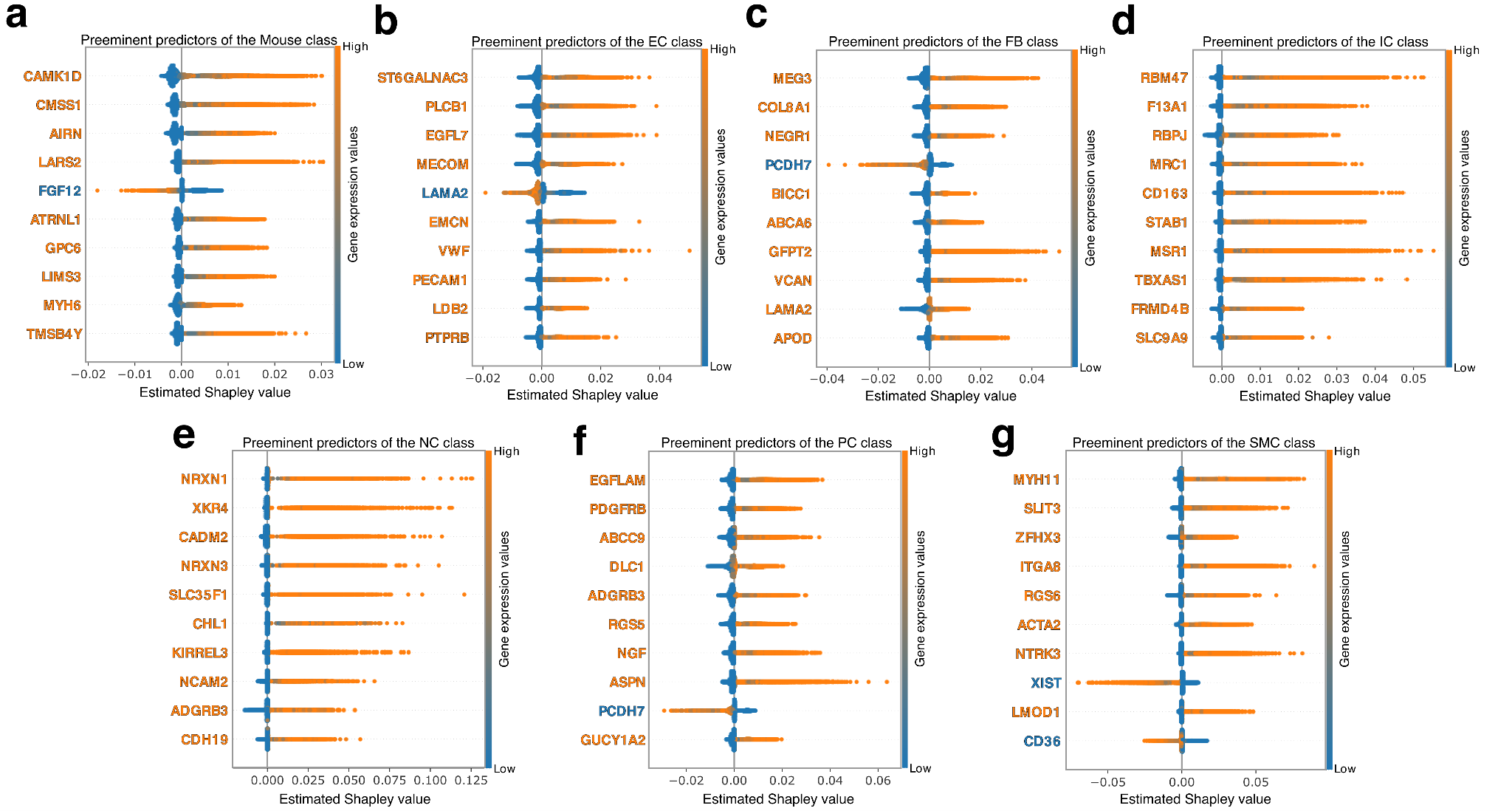


**Extended Data Fig. 5: XAI analysis of the classifier nodes for mouse and the remaining cell types. a-g**, Most influential predictors contributing to the **a)** mouse class, **b)** endothelial cell (EC) class, **c)** fibroblast (FB) class, **d)** immune cell (IC) class, **e)** neuronal cell (NC) class, **f)** pericyte (PC) class and **g)** smooth muscle cell (SMC) class. High expression values in the data are highlighted in orange while low expressions are blue. Depending on the Shapley values, the levels of expression can therefore be associated with a higher or lower prediction result for a given gene. An orange gene name indicates a positive correlation between expression and prediction for that class, while a blue gene name indicates a negative correlation.


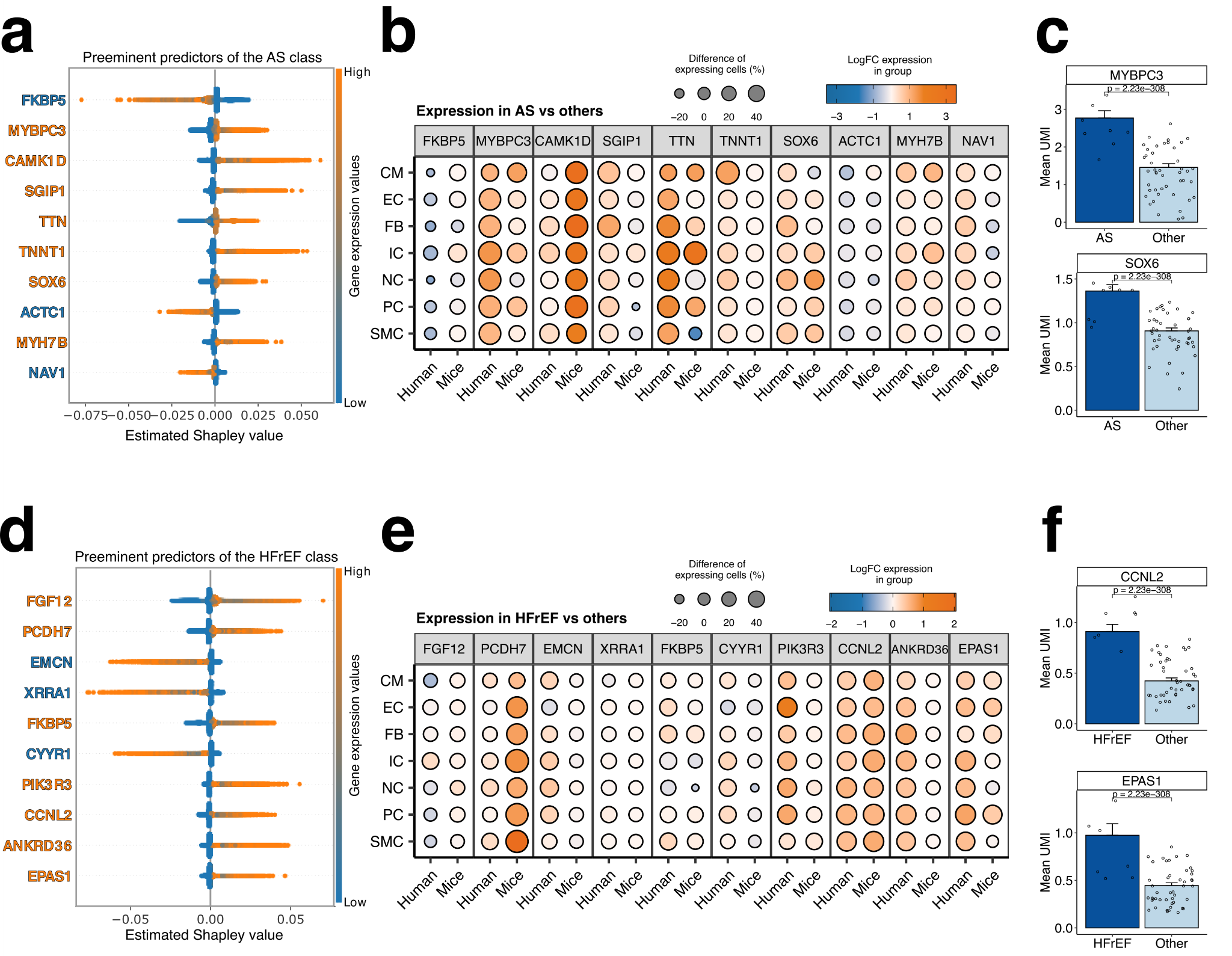


**Extended Data Fig.6: XAI analysis for heart failures caused by aortic stenosis (AS) and heart failure with reduced ejection fraction (HFrEF). a,d,** Ten most contributing predictors visualized by a beeswarm plot for **a)** aortic stenosis and **d)** HFrEF cells. An orange gene name indicates a positive correlation between expression and prediction for that class, while a blue gene name indicates a negative correlation similar as in Figure 3. **b,e,** Dot plot illustrating the log fold change in gene expression and the percentage change of cells expressing the indicated genes. Panels represent the following **b)** AS predictors by cell type across human and mouse and **e)** HFrEF predictors by cell type and species. **c,f,** Averaged unique molecular identifiers (UMI) for two selected genes for **c)** aortic stenosis against the remaining conditions and **f)** HFrEF cells against the remaining conditions. Statistical significance was determined using the Wilcoxon test on single cell expressions.


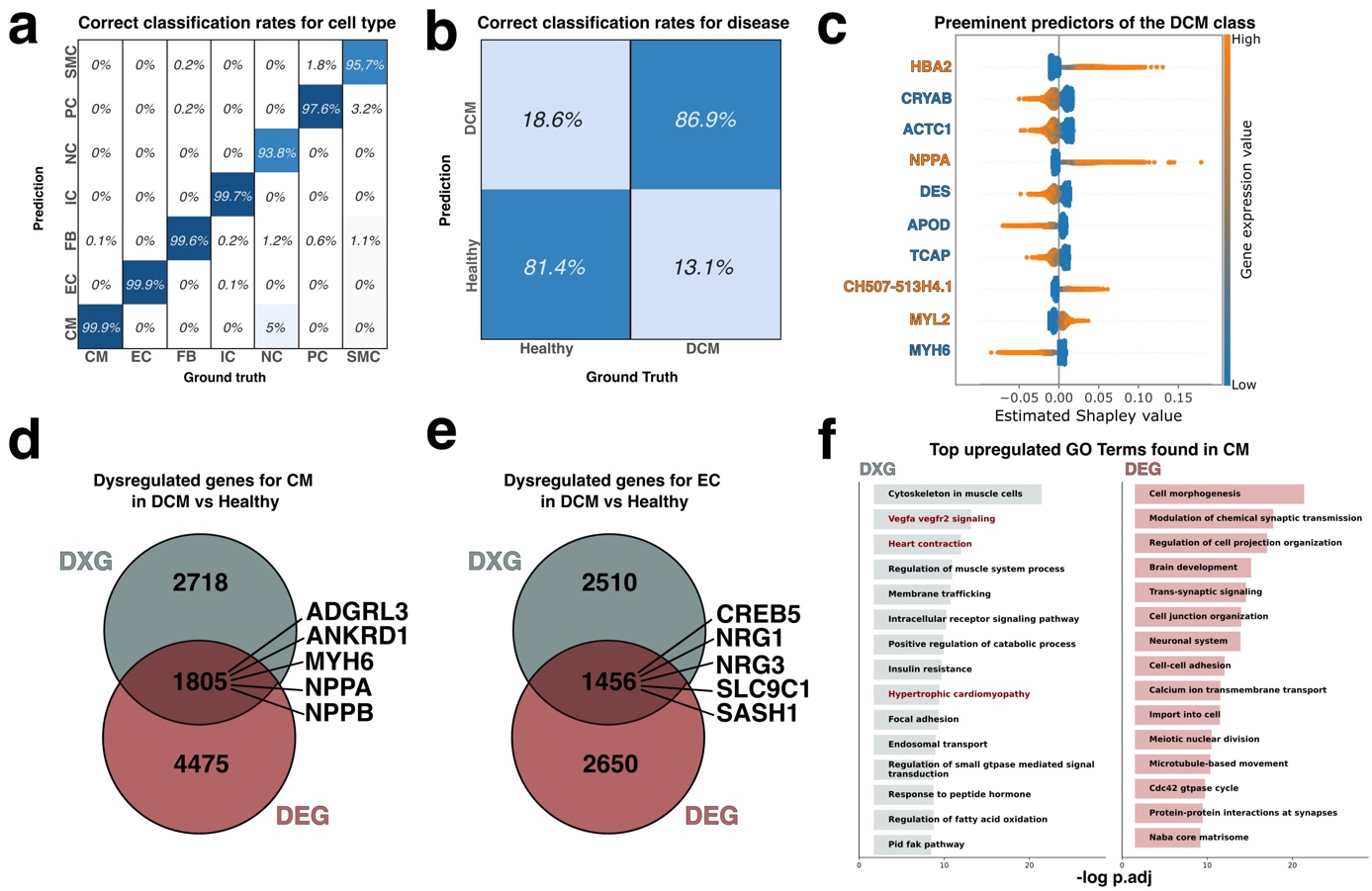


**Extended Data Fig. 7: DXG analysis on independent dataset from Koenig et al.**[^23^](https://paperpile.com/c/8Lvr7k/a9FX6) **comprising 27 healthy and 18 DCM patients** **a-b,** Confusion matrix of correct classification rates averaged for **a)** cell type and **b)** disease. **c,** Ten most contributing predictors visualised by a beeswarm plot for DCM. **d-e,** Comparison of the overlap of dysregulated genes identified in **d)** CM under the DCM condition versus the Healthy condition and in **e)** EC. DXGs were calculated by applying a t-test to Z-transformed Shapley values, while DEGs were obtained from the published study, with genes described in the study being highlighted. **f,** Top ten upregulated GO terms for CM under the DCM condition ranked by logarithmised Benjamini-Hochberg corrected p-values, based on DXG lists (left) and expression values (right).
